# A longitudinal evaluation of the impact of a polylactic acid injection therapy on health related quality of life amongst HIV patients treated with anti-retroviral agents under real conditions of use

**DOI:** 10.1186/1471-2334-13-92

**Published:** 2013-02-20

**Authors:** Martin Duracinsky, Pascale Leclercq, Andrew Richard Armstrong, Marc Dolivo, Frédéric Mouly, Olivier Chassany

**Affiliations:** 1 AP-HP, Bicêtre hospital, Infectious Diseases, Kremlin-Bicêtre, France; 2 University hospital, Infectious Diseases, Grenoble, France; 3 AP-HP, Saint-Louis hospital, Department of Clinical Research: Patient Reported Outcomes Unit, University Paris-Diderot, Paris, France; 4 Dermatologist, Paris, France

## Abstract

**Background:**

Many HIV patients receiving antiretroviral treatment develop lipodystrophy. NEW-FILL^®^ is a polylactic acid injected to treat facial lipoatrophy. The objectives of this study were to describe (1) change in quality of life (QoL) of HIV patients treated with NEW-FILL^®^ in the management of facial lipoatrophy; (2) efficacy of NEW-FILL^®^ using facial photographs and (3) a patient-reported “Overall Treatment Effect” (OTE) scale; and (4) safety of NEW-FILL^®^.

**Methods:**

Doctors from 13 treatment centres recruited 230 HIV patients to receive up to 5 sessions of NEW-FILL^®^ injections. Patients self-reported QoL with the ABCD questionnaire before the first set of injections, at 2 months and at 12 to 18 months after the last session of injections. Efficacy was evaluated at each interval through photographs and OTE scale. Safety was evaluated via Case Report Form (CRF) data.

**Results:**

64.4% of patients reported QoL improvements of >10% at 2 months, and 58.8% at 12–18 months. Lipoatrophy grades improved at each visit (“no lipoatrophy” or “limited lipoatrophy”: 20.3% at inclusion, 77.4% at 2 months, 58.4% at 12–18 months). Average OTE scores of 5.3 and 5.0 at 2 and 12–18 months indicated “moderate improvement”. Minimum Important Difference (MID) in QoL score was 7.1 points at 2 months; 7.4 points at 12–18 months. For 911 injection sessions performed, 3.4% resulted in “immediate” adverse events, 7% in “non-immediate” events, and 1.7% in “other” events.

**Conclusions:**

Improvements to quality of life and diminished lipoatrophy visibility were observed in the months immediately following NEW-FILL^®^ treatment and were maintained 12–18 months post-treatment. Most adverse events were mild and transient. ABCD MID thresholds provide clinicians with means to assess the impact of lipoatrophy therapies on QoL.

## Background

The use of antiretroviral agents for treatment of HIV infection has made it possible to considerably improve morbidity and mortality associated with HIV infection. But their use exposes patients to adverse events in the medium and long term. Lipodystrophy syndrome is one of the main effects in the long term observed in patients treated with antiretroviral agents. Lipodystrophy is probably the result of the synergistic effect of several classes of medicinal products because some protease inhibitors (PI) inhibit adiposity differentiation and induce insulin resistance while some nucleoside inhibitors (NI) produce lipolysis and, under some conditions, apoptosis of adipocytes. The difference in sensitivity of peripheral and central adipocytes to these compounds may partly explain the redistribution of fat, keeping in mind that other mechanisms are probably involved [1].

Many cross-sectional studies have demonstrated the prevalence of lipodystrophy ranging from 18 to 80% in patients who received antiretroviral therapy [2-5]. It is estimated that about 50% of patients develop lipodystrophy after one to two years of exposure to multiple drug therapy including a protease inhibiter (PI) [6].

The psychological impact of lipodystrophy is substantive [7,8]. Physical changes, especially facial atrophy, stigmatise patients and affect compliance with therapy [9] and patient’s quality of life [8,10]. The success of antiretroviral therapy to a large extent is based on patient compliance with therapy [11]. The Aproco [9] cohort clearly demonstrated the relationship between lipodystrophy and non-compliance with therapy. It is therefore important to improve the impact of therapies on quality of life and consequently on compliance.

One method of managing facial lipoatrophy is to inject filler products. Two main types can be differentiated, permanent filler products and absorbable filler products. NEW-FILL^®^ or L-Polylactic Acid is an absorbable filler product whose purpose is to produce progressive thickening of the dermis by deep intradermal injection. Its slow absorbability gives it an extended duration of action [12].

In France, L-polylactic acid (NEW-FILL^®^) is a medical device which obtained a favourable opinion from the Commission for Evaluation of Products and Services (CEPP) [13] for reimbursement in correction of facial lipoatrophy in HIV-positive patients treated with antiretroviral therapies. The impact of this treatment on patients’ quality of life has not previously been addressed.

The objectives of this study were four-fold: [1]. The primary objective was to describe the impact of NEW-FILL^®^ on the quality of life of patients treated with antiretroviral agents, using the lipodystrophy-specific ABCD quality of life self-questionnaire [14,15]. The secondary objectives of this study were: [2] to describe the efficacy of NEW-FILL^®^ based on photographs taken before the first injection, 2 months after the last injection session, and then, if applicable, 12 and 18 months after the last injection session; [3] to describe overall efficacy of treatment with an “Overall Treatment Effect” (OTE) scale [16]; [4] to contribute further data regarding the safety of NEW-FILL^®^ in the short, medium and long term.

## Method

### Design

This was an observational, longitudinal, multicenter, open label study performed in France. The study followed patients during their usual care routine. Additional information pertaining to the study was asked for during usual care visits. It did not alter patients’ usual medical management nor affect their physical or psychological integrity nor require specific monitoring visits. A participation consent form was signed jointly by patient and investigating doctor. The study was approved by the Paris Ile de France 4 Ethics Committee, Paris, France. The study was conducted in compliance with the Helsinki Declaration [17], with the French Data Processing and Liberties Law no 78.17 of 06.01.1978, received a favourable opinion from the CCTIRS (Consultative Committee on Data Processing in Research in the field of Health), and was authorised by CNIL (National Commission on Data Processing and Liberties).

### Schedule

The first patient was included in April 2006 and the last in April 2007. Final follow-up with the last patient occurred in February 2009.

### Recruitment of doctors and patients

Thirteen ‘investigating’ doctors were recruited from 13 centres where NEW-FILL^®^ treatment was provided. Investigating doctors were recruited from a list of all doctors trained in the injection technique who had treated at least one patient with NEW-FILL^®^. A total of 200 patients were recruited from the 13 centres and comprised the first 20 patients from each centre agreeing to participate in the study and who met eligibility criteria during a 6 month period.

### Inclusion/non-inclusion criteria

Inclusion criteria were: men or women at least 18 years of age, HIV-seropositive, treated with antiretroviral therapy and for whom the doctor freely decided to use NEW-FILL^®^ to correct facial lipoatrophy, who agreed to participate in the study and who signed the consent form, with an adequate level of education to be able to fill out alone the self-questionnaires on evaluation of quality of life and overall efficacy of treatment. Non-inclusion criteria were: patients participating in another study with a similar product.

### Data collection

Patients completed the ABCD quality of life questionnaire before the first injection and then 2 months after the last session of injections and, if applicable, 12 to 18 months after the last session of injections. Concomitantly, the Overall Treatment Effect (OTE) scale was completed by patients 2 months after the last session of injections and 12 to 18 months after the last session of injections, if applicable*. Comparison of ABCD and OTE scores allowed calculation of the minimal important difference in overall ABCD quality of life score.

*As it was an observational study the 12/18 months evaluation took place only if the patient returned to a visit during this period in the setting of usual management

An expert committee (2 dermatologists, 1 plastic surgeon) graded photographs for lipoatrophy presence before the 1^st^ injection, 2 months after the last session of injections and, if applicable, 12 to 18 months after the last session of injections. Safety was evaluated using side effect or adverse event data obtained from the Case Report Forms (CRF).

The *Assessment of Body Change and Distress (ABCD) questionnaire*[14] consist of three parts. Lipodystrophy: Six items concern the presence/absence of lipodystrophy in 6 areas of the body. The score is calculated by adding up the total number of responses (0 or 1) to the 6 items (0= no area affected 6 = 6 areas affected). Satisfaction: One 5-point item investigates overall satisfaction with body appearance (from 1 = very dissatisfied to 5 = very satisfied). Quality of life: 21 items spread across four dimensions evaluate the impact of lipodystrophy on the patient’s life; Dimension A: Control and adjustment to the illness, satisfaction with body appearance (9 items); Dimension B: Psychological, social and relational impact (7 items); Dimension C: Fear of the future (3 items); Dimension D: Impact of treatment (2 items). Note that this French ABCD questionnaire contains an additional item concerning surgery/aesthetic correction of lipodystrophy in Dimension B. Quality of life items were summed to form an overall QoL score (Cronbach’s alpha 0.89), t-transformed to range from 0 = worst possible QoL to 100 = best possible QoL.

*Lipoatrophy grading system*[18]*.* For each patient, photographic slides (each containing 3 views: 1 front, 1 right side, 1 left side) were submitted to a committee of 3 experts in random order, blinded (before treatment/after treatment or after treatment/before treatment), and without indication of the injection site. The experts rated the presence of lipoatrophy in each photo according to a grading system which differentiates 4 stages of lipoatrophy: Grade 1: corresponds to limited lipoatrophy, localised to the face. The appearance is almost normal. Grade 2: corresponds to deeper lipoatrophy. Some muscles begin to be visible, in particular the zygomatic. Grade 3: the affected area is no longer localised solely to the cheeks but is also deeper and wider. The muscles are increasingly visible. Grade 4: the most serious condition, also shows damage to the orbits. In this study, Grade 0 (no lipoatrophy) was added to the grading system. Photographs of patients were classified into one of 5 stages before treatment and then again classified at the visit at 2 months after treatment and at 12–18 months after treatment.

The *Overall Treatment Effect (OTE) scale*[16] is a global rating of change scale. It comprises a single item on which patients rate the overall effect of treatment on their condition on a 15-point scale, where −7 = condition greatly worsened, 0 = no change, +7 = condition greatly improved.

*Adverse events* likely to be associated with injection of NEW-FILL^®^ were classified as follows: “immediate” adverse events, such as bleeding, redness at the injection site, pain, ecchymosis and facial oedema. These coincided to time of injection. “Non-immediate” adverse events, such as nodules and/or indurated areas, granulomas, inflammation, discolouration, allergic reactions, coetaneous hypertrophy and atrophy. Clinically, they were of late onset. They are difficult to attribute to a particular session of injections because they can result from successive effect of several sessions of injections. When an adverse event was not an expected event (in conformity with the product leaflet), it was classified as “other” and entered according to the doctors verbatim.

### Statistical methods

Statistical analyses were performed with SAS software, version 8.2 [19]. Data were described in terms of sample size, mean, standard error, percentage by modality, response, and number of missing data, pre- (time 1) and post-treatment (time 2: 2 months; time 3: 12–18 months). Evaluation of quality of life was performed based on a score calculated according to recommendations of the ABCD questionnaire. Wilcoxon t-tests were used to determine the significance of within-group changes over time in QoL component scores between the inclusion or pre-treatment visit (time 1) and post-treatment visits (time 2, time 3). Determination of the minimal important difference in quality of life was derived from calculation of the pre-treatment (time 1) and post-treatment (time 2, time 3) change in QoL score that corresponded with an OTE rating [2,3] of a condition ‘a little improved’ or ‘somewhat improved’. Evaluation of photographs pre- (time 1) and post-treatment (times 2,3) were performed by a committee of experts according to the scale for classification of lipoatrophy. Frequency of reported side effects were calculated.

## Results

Participant data collected from 230 patients between April 2006 and February 2009 were analysed. Patient characteristics are shown in Table 1. Patients were 47.4 years of age on average. Eighty-seven percent of participants were male. The average duration of HIV infection was around 16 years and time since initiation of anti-retroviral therapy was 11.2 years. Time since diagnosis of facial lipoatrophy was 5 years. Previous treatment of facial lipoatrophy was found in 39.4% of patients (NEW-FILL^®^ in 75% of cases). CD4 count was greater than or equal to 350 CD4/mm3 in 74.5% of patients; almost 85% of patients had a viral load of less than 400 copies/ml and almost 60% had a viral load of less than 50 copies/ml. The most frequently observed combinations of antiretroviral agents were 2 Nucleosid Reverse-Transcriptase Inhibitors (NRTI) + 1 Protease Inhibitor (PI) (36.1%) and 2 NRTIs +1 non-NNRTI (Non-Nucleoside Inhibitor; 21.3%). Demographic characteristics of patients evaluated at 12–18 months and of patients lost to follow-up were very similar (Mean age: 48.2 years vs. 46,7 years respectively, % men: 84.9% vs. 88.7% respectively, mean body mass index (BMI): 21.4 kg/m^2^ vs. 21.7 kg/m^2^ respectively).

**Table 1:** Patient characteristics

	**Total population**	**Quality of life population**	**Photographed population**
	**N=230**	**N=197**	**N=177**
Age (years)	47.42 (8.40)	47.85 (8.47)	48.09 (8.78)
Sex (Male)	87.0%	86.8%	87.6%
Body Mass Index	21.59 (2.56)	21.62 (2.50)	21.63 (2.45)
Duration of HIV seropositivity (years)	16.00 (4.37)	16.10 (4.29)	16.11 (4.35)
CD4 count (CD4/mm^3^)	543 (299)	547 (288)	551 (306)
Viral load (< 400 copies/ml)	84.9%	84.7%	84.0%
Time since antiretroviral therapy initiated (years)	11.55 (4.02)	11.69 (3.90)	11.74 (3.98)

Note: Means are provided for continuous variables with standard error of measurement (SEM) in parentheses.

Data on 911 sessions of injections were collected on the 230 patients, i.e. a mean of 4 sessions of injections per patient. Over half of the patients (51.3%) had 5 sessions of injections of NEW-FILL^®^.

Injection sessions most often involved the cheeks (98.9%) and temples (37.7%). Other facial areas were a rarer focus (7.7%). One hundred and ninety-seven patients had an evaluable ABCD questionnaire at inclusion (at least 50% of items completed), an evaluable ABCD questionnaire at one monitoring visit and an OTE questionnaire filled out at the same monitoring visit. One hundred and seventy-seven patients had a photograph considered evaluable at inclusion and at the monitoring visit at 2 months. A photograph was considered evaluable if it could be classified by the expert committee according to the lipoatrophy grading system. Patients for whom the course of NEW-FILL^®^ was not followed by a monitoring visit at 12–18 months after the last injection session or whose evaluation visit at 12–18 months was not evaluable were considered as lost to follow-up. Of the 230 patients included, 124 were lost to follow-up.

The first and primary objective of this study was to describe the change in quality of life of patients treated with NEW-FILL^®^ in the setting of management of facial lipoatrophy in HIV-seropositive subjects treated with antiretroviral agents. Results of the study showed significant improvement in all mean scores on the ABCD questionnaire measured between inclusion and monitoring visits at 2 months and 12 to 18 months (see Table 2). Between the inclusion visit and 2 month visit, 64.4% of patients had an improvement in their overall quality of life score (QoL) of at least 10% and in addition, 27,8% of patients had an improvement in their QoL score of at least 50%. Between inclusion visit and 12–18 month visit, 58.8% of patients improved their overall quality of life score by at least 10%, and 22.7% had an improvement of at least 50%.

**Table 2:** ABCD scores at inclusion visit and at each monitoring visit – Quality of life population

	**Inclusion visit**	**Monitoring visit 2 months**	**Monitoring visit 12–18 months**
	**N=197**	**N=191**	**N=97**
Lipodystrophy score	3.31 (1.14)	2.23 (1.72)*	2.14 (1.61)*
Overall satisfaction with body image	2.56 (0.91)	3.30 (1.03)*	3.13 (1.01)*
Dimension A: Control and adjustment to illness, satisfaction with body image	53.13 (21.24)	67.84 (23.73)*	66.19 (20.04)*
Dimension B: Psychological, social and relational impact	52.13 (22.85)	67.37 (22.84)*	65.08 (18.62)*
Dimension C: Fear of the future	60.00 (24.04)	70.53 (24.08)*	65.08 (18.62) ^†^
Dimension D: Impact of treatment	83.74 (21.09)	87.63 (19.74)*	86.60 (19.87) ^†^
Overall ABCD quality of life score	56.55 (18.97)	69.93 (20.53)*	67.92 (16.91)*

* Comparison with score at inclusion: Wilcoxon Test, p = <0.001.

† Comparison with score at inclusion: Wilcoxon Test, p = <0.05.

Means provided with standard error of measurement (SEM) in parentheses.

Scores for Dimensions A to D are t-transformed where 0 = worst and 100 = best possible QoL.

Overall quality of life scores of patients evaluated at 12–18 months and of patients lost to follow-up were similar at inclusion (57.2 vs. 55.9 respectively) and at the monitoring visit at 2 months (70.0 vs. 69.9 respectively). Lipodystrophy scores of patients evaluated at 12–18 months and patients lost to follow-up were similar at the inclusion visit on average (mean score of 3.3 in the 2 sub-groups). At the monitoring visit at 2 months, the lipodystrophy score of patients evaluated at 12–18 months appeared as slightly higher than that of patients lost to follow-up on average (mean scores of 2.3 and 2.1 respectively). Patients lost to follow-up after the visit at 2 months, therefore, were not patients who said that their condition worsened. The satisfaction score at the inclusion visit for patients lost to follow-up was slightly higher than that of patients evaluated at 12–18 months on average (2.7 vs. 2.5 respectively). At monitoring visit at 2 months, the satisfaction scores of patients evaluated at 12–18 months and patients lost to follow-up were similar on average (3.3 for the 2 sub-groups). The patients lost to follow-up after the visit at 2 months therefore were not dissatisfied patients.

The second objective of the study was to describe efficacy of NEW-FILL^®^ based on photographs taken before the first injection, 2 months after the last session if injections, and then, if applicable, 12 to 18 months after the last session of injections.

Analysis of photographs taken at the inclusion visit and at monitoring visits of 2 months and 12–18 months was performed according to the lipoatrophy grading system (see Table 3). The results of the study demonstrated a significant improvement in grade of lipoatrophy between the inclusion visit and monitoring visit (Grades 0 or 1: 20.3% at inclusion, 77.4% at 2 months, 58.4% at the 12–18 months). Furthermore, 55.4% of patients saw their lipoatrophy decrease by one grade between inclusion visit and the monitoring visit at 2 months, 31.6% by two grades. Similarly, 46.7% of patients saw their lipoatrophy decrease by one grade between the inclusion visit and the monitoring visit at 12–18 months, 23.3% of patients by two grades.

**Table 3:** Facial lipoatrophy (FL) grades based on photographs at inclusion visit and at each monitoring visit

	**Inclusion visit**	**Monitoring visit 2 months**	**Monitoring visit 12–18 months**
	**N=177**	**N=177**	**N=90**
Grade			
0 No visible FL	1.1%	34.5%	21.3%
1	19.2%	42.9%	37.1%
2	48.0%	16.9%	30.3%
3	27.1%	5.6%	11.2%
4	4.5%	0.0%	0.0%

Grades range from 0 (no FL), 1 (Slight localised facial lipoatrophy), 2 (Longer and deeper atrophy with start of appearance of facial muscles), 3 (Deeper and wider area of atrophy, facial muscles clearly apparent), 4 (Lipoatrophy covering a wide area, with extension to the eye orbits and facial skin resting directly on the muscles).

The third objective of the study was to describe overall efficacy of treatment with the OTE questionnaire (scored −1 to −7 in the case of a worsened condition, 0 in case of no change and +1 to +7 in the case of improvement). The average OTE questionnaire scores at monitoring visits 2 months and 12–18 months were 5.3 ± 1.5 and 5.0 ± 1.9 respectively (with ‘5’ indicating a moderate improvement in condition due to treatment). No patient mentioned deterioration. The minimum reported score was 0 (“no change”).

Table 4 shows changes in ABCD QoL scores between inclusion and monitoring visits that correspond with clinically important difference thresholds. OTE data indicated that the minimal clinically important change in the overall ABCD QoL score was 7.1 points between inclusion and monitoring visit at 2 months and 7.4 points for the monitoring visit at 12–18 months. Improvements of this magnitude in QoL scores corresponded with OTE ratings of 2 or 3 points reflecting a condition ‘a little better’ or ‘somewhat better’. A moderate important change (4 or 5 point OTE rating) for the QoL score was 11.1 for the monitoring visit at 2 months and 5.9 for monitoring visit at 12–18 months. The maximal important change (6 or 7 point OTE rating) for the quality of life score was 16.1 on average for the monitoring visit of 2 months and 14.2 for monitoring visit at 12–18 months.

**Table 4:** Minimal, moderate and maximal important changes in ABCD Quality of Life scores between inclusion and monitoring visits

	**Minimal important difference**		**Moderate important difference**		**Maximal important difference**	
	**2 month visit**	**12-18 month visit**	**2 month visit**	**12-18 month visit**	**2 month visit**	**12-18 month visit**
Change in ABCD QoL score	7.11	7.38	11.10	5.89	16.08	14.18
	(9.74)	(12.88)	(18.00)	(13.80)	(18.56)	(15.51)

Note: Means provided with SEM in parentheses.

The fourth objective of the study was to describe safety of NEW-FILL^®^ in the short, medium, and long-term. The occurrence of at least one adverse event whatever the type was reported for 34 patients (14.8%). 19 patients (8.3%) had at least one “immediate” adverse event. Out of 911 injection sessions performed, at least 1 “immediate” adverse event was reported for 31 injection sessions (3.4%). Among these “immediate” adverse events, ecchymosis (1.9% of injection sessions), pain (1% of injection sessions) and redness at the injection site (0.5% of injection sessions) were the most common effects.

At least one “non-immediate” adverse event was reported by 16 patients (7%). The most frequently reported “non-immediate” adverse events were nodules and/or indurated areas (3.9% of patients); the occurrence of granulomas was reported in 2 patients (0.9%). Median times of occurrence of these adverse events after start of the course of therapy were 75 days for nodules and/or indurated areas and 40 days for granulomas. In agreement with legislation on medical device vigilance, as a result of administration of corrective therapy, one of the cases of granuloma was reported as a “serious” adverse event. Granulomas regressed in 2 cases of occurrence. Regression of nodules and/or indurated areas, inflammation and coetaneous hypertrophy was reported in 33.3%, 66.7% and 50.0% of cases respectively. Four patients (1.7%) were the subject of reporting of an “other” adverse event. In all cases, it involved malaise which occurred on the day of injection.

## Discussion

This study investigated [1] changes in patient perceptions of quality of life following lipoatrophy treatment with NEW-FILL^®^, [2] the efficacy of NEW-FILL^®^ based on photographs taken before and after treatment [3], patients perception of overall treatment effect, and [4] safety of NEW-FILL^®^ in terms of adverse treatment-related events.

Patients reported significant improvements in all aspects of quality of life following treatment. Concerns about HIV treatment reduced, as did fears for the future. Psychological, social and relational aspects of quality of life were especially improved, as was satisfaction with body appearance. Heightened scores on the latter QoL dimensions demonstrate the value of having used a condition-specific QoL instrument to assess the impact of condition-targeted treatment. These dimensions, encompassing intrapersonal and interpersonal stigma and appearance, are most indicative of lipoatrophy QoL effects [12] while HIV treatment concerns and future fears relate to HIV QoL more generally [10,20,21]. Similar studies using generic QoL measures such as the SF-36 have sometimes failed to find significant treatment gains [22], while others have failed to show QoL differences between patients with and without lipodystrophy [4]. On the other hand, studies using the ABCD have reported comparable results [23,24].

Patient-reported QoL improvements corresponded with doctor ratings of lipoatrophy reductions in facial disfigurement and musculature visibility observed in patient photos at three time intervals. Approximately half of patients saw their lipoatrophy decrease by at least one grade, and a quarter decreased by two grades between visits at 2 months and 12–18 months. Previous studies of the impact of absorbable injection therapies [e.g. polyactic acid (PLA)] have also shown convergence between patient-reported QoL improvements and clinician-reported improvements in lipoatrophy [25]. This convergence of views differs from some studies in which surgery has been the treatment of choice, and in which clinicians reported surgical success while patients reported significant QoL issues arising from post-surgery complications. Moreover, comparative studies have shown greater patient-reported QoL gains resulting from reabsorbable (PLA) and non-reabsorbable [e.g. polyacrylamide hydrogel (PAAG)] filler injections when compared to autologous fat transfer (AFT) surgery [23]. This speaks to the value of including both patient and doctor perspectives as a method of evaluating condition improvement, and to the use of filler injection techniques as the treatment of choice.

Patient ratings of the overall treatment effect were positive in all cases, with most declaring a moderate improvement. No patient mentioned deterioration in condition as a result of treatment. A within subject change of 2–3 points in overall treatment effect (‘a little improved’ or ‘somewhat improved’) equated with approximately 7 points change in ABCD quality of life score, indicating the minimal clinically important difference threshold for the ABCD QoL scale. Thresholds were determined for changes in ABCD QoL score for short and longer-term follow-up assessment (2 and 18 months), and for determining moderate and major important differences. With this knowledge, treating health professionals can easily and effectively monitor the magnitude of impact of a lipoatrophy treatment without the interpretive ambiguity typically associated with other QoL instruments. Furthermore, clinicians need not rely upon expensive and complicated diagnostic procedures and equipment to assess the need for patient intervention.

In this study, the primary criterion to assess the impact of lipoatrophy was the subjective perception of patients. It can be seen that this criterion is extremely useful in the clinical assessment of need for lipoatrophy correction, and may be a better indicator of treatment need than medical assessment procedures commonly used in clinical trials such as DEXA scan, bioimpedance or sonography. For instance low sensitivity poses a problem for the use of sonography to detect LD in the clinical routine as a single exam [24].

Treatment safety data concur with the findings of previous studies [26-29]. Immediate adverse events were reported by around 8% of patients, with ecchymosis, pain and skin redness being the most common. Non-immediate events affected around 7% of patients, with nodules and inflammation being the most frequently reported. These essentially mild and transient events, coupled with significant QoL gains and visible lipoatrophy improvements, speak to the value of preferencing cosmetic filler therapy over surgery for which there are greater immediate risks such as disfigurement, expertise required, lack of long-term safety data, and limits to whom surgery is applicable [22].

In terms of clinical value, compliance with anti-retroviral therapy may well improve with the use of NEW-FILL^®^. The success of ART is largely based on patient compliance with therapy [11], and lipodystrophy diminishes compliance [9]. As shown here, improvements to lipoatrophy occurred following NEW-FILL^®^ treatment in ways that were meaningful and recognisable to both doctor and patient.

### Limitations

One limitation is the observational design of the study. Improvement in QoL may not solely be attributed to treatment. However, spontaneous improvement of lipoatrophy does not occur. Consistent patient improvements observed in the photographs add weight to the validity of the results. Moreover, the efficacy of polyactic acid when compared to other treatments has been shown in several previous comparative studies [22,23,27-29]. A second limitation is the loss of patients to followup, though data available indicated that those lost had similar demographics and treatment outcomes in the early stages to those retained in the later stage. Thirdly, most participants were male hence the findings best represent the treatment experience of men rather than women. A fourth limitation is the use of a single-item measure to establish MID. The reliability of a single item global measure of change is low, and it is strongly influenced by the present state and only weekly by the initial state. Others studies have used multiple item measures, effect size, and combined clinical-patient ratings of overall treatment effect for MID calculation.

## Conclusions

In summary, the results demonstrated an improvement in quality of life scores as well as a favourable change in visual grade of lipoatrophy between inclusion visit and monitoring visit at 2 months post-NEW-FILL^®^ treatment. These improvements were maintained 12–18 months after the end of the course of therapy. The majority of adverse events reported were mild and transient. The occurrence of nodules or granulomas remained infrequent.

## Competing interests

This study was sponsored by Sanofi-Aventis France. With the exception of Dr AR Armstrong, the authors received consulting fees from Sanofi-Aventis France.

## Authors’ contribution

MDu – participated substantially in the conception and design of the study, development of the quality of life measure, analyses and interpretation of data, drafting and revision of the manuscript. PL - participated substantially in the design of the study, interpretation of data, drafting and revision of the manuscript. AA – participated substantially in the analyses and interpretation of data, drafting and revision of the manuscript. MDo – participated substantially in the design of the study, acquisition of data, drafting and revision of the manuscript. FM – participated substantially in the design of the study, acquisition of data, drafting and revision of the manuscript. OC - participated substantially in the conception and design of the study, development of the quality of life measure, interpretation of data, drafting and revision of the manuscript. All authors read and approved the final manuscript.

## Pre-publication history

The pre-publication history for this paper can be accessed here:

http://www.biomedcentral.com/1471-2334/13/92/prepub
